# Long-Term Post–COVID-19 Health and Psychosocial Effects and Coping Resources Among Survivors of Severe and Critical COVID-19 in Central and Eastern Europe: Protocol for an International Qualitative Study

**DOI:** 10.2196/57596

**Published:** 2024-09-30

**Authors:** Anna Alexandrova-Karamanova, Anita Lauri Korajlija, Peter Halama, Adriana Baban

**Affiliations:** 1 Department of Psychology Institute for Population and Human Studies Bulgarian Academy of Sciences Sofia Bulgaria; 2 Department of Psychology Faculty of Humanities and Social Sciences University of Zagreb Zagreb Croatia; 3 Centre of Social and Psychological Sciences Slovak Academy of Sciences Bratislava Slovakia; 4 Department of Psychology Babes-Bolyai University Cluj-Napoca Romania

**Keywords:** COVID-19 survivors, severe COVID-19, COVID-19 hospitalization, long-term COVID-19 effects, post–COVID-19 condition, posttraumatic growth, coping resources, qualitative, international, Central and Eastern Europe

## Abstract

**Background:**

There is a strong need to determine pandemic and postpandemic challenges and effects at the individual, family, community, and societal levels. Post–COVID-19 health and psychosocial effects have long-lasting impacts on the physical and mental health and quality of life of a large proportion of survivors, especially survivors of severe and critical COVID-19, extending beyond the end of the pandemic. While research has mostly focused on the negative short- and long-term effects of COVID-19, few studies have examined the positive effects of the pandemic, such as posttraumatic growth. It is essential to study both negative and positive long-term post–COVID-19 effects and to acknowledge the role of the resources available to the individual to cope with stress and trauma. This knowledge is especially needed in understudied regions hit hard by the pandemic, such as the region of Central and Eastern Europe. A qualitative approach could provide unique insights into the subjective perspectives of survivors on their experiences with severe COVID-19 disease and its lingering impact on their lives.

**Objective:**

The aim of the study is to qualitatively explore the experiences of adult survivors of severe or critical COVID-19 throughout the acute and postacute period in 5 Central and Eastern European countries (Bulgaria, Slovakia, Croatia, Romania, and Poland); gain insight into negative (post–COVID-19 condition and quality of life) and positive (posttraumatic growth) long-term post–COVID effects; and understand the role of survivors’ personal, social, and other coping resources and local sociocultural context and epidemic-related situations.

**Methods:**

This is a qualitative thematic analysis study with an experiential reflexive perspective and inductive orientation. The analytical approach involves 2-stage data analysis: national analyses in stage 1 and international analysis in stage 2. Data are collected from adult survivors of severe and critical COVID-19 through in-depth semistructured interviews conducted in the period after hospital discharge.

**Results:**

As of the publication of this paper, data collection is complete. The total international sample includes 151 survivors of severe and critical COVID-19: Bulgaria (n=33, 21.8%), Slovakia (n=30, 19.9%), Croatia (n=30, 19.9%), Romania (n=30, 19.9%), and Poland (n=28, 18.5%). National-level qualitative thematic analysis is currently underway, and several papers based on national results have been published. Cross-national analysis has started in 2024. The results will be submitted for publication in the third and fourth quarters of 2024.

**Conclusions:**

This research emphasizes the importance of a deeper understanding of the ongoing health and psychosocial challenges survivors face and what helps them cope with these challenges and, in some cases, thrive. It has implications for informing holistic care and improving the health and psychosocial outcomes of survivors of COVID-19 and will be crucial for evaluating the overall impact and multifaceted implications of the pandemic and for informing future pandemic preparedness.

**International Registered Report Identifier (IRRID):**

DERR1-10.2196/57596

## Introduction

### Long-Term Negative Health and Psychosocial Effects of COVID-19

The COVID-19 pandemic has become a major health crisis across the world. The new SARS-CoV-2 virus has caused over 774 million confirmed cases and over 7 million deaths globally [1]. As the pandemic progressed, in addition to the acute disease, a prolonged form has been identified, known as post–COVID-19 condition (or long COVID, post–COVID-19 syndrome, and postacute sequelae of SARS-CoV-2 infection [PASC]), with a global estimated prevalence of 43% [2]. The condition is characterized by long-term symptoms and complications that continue or develop after acute COVID-19. The World Health Organization (WHO) defines the post–COVID-19 condition as occurring usually 3 months from the onset of COVID-19 with symptoms that last for at least 2 months. Symptoms may be new onset, following initial recovery from an acute COVID-19 episode, or persist from the initial illness and generally have an impact on everyday functioning. Symptoms may also fluctuate or relapse over time [3]. Clinical guidelines in the United Kingdom and the United States define the prolonged form of COVID-19 as symptoms ongoing for 4 weeks or more [4,5]. A variety of long-term, patient-reported symptoms have been identified in adult survivors of COVID-19, with over 200 different symptoms reported across 10 organ systems [6]. Commonly reported symptoms include fatigue, shortness of breath, brain fog (concentration problems, cognitive dysfunction), headache, dizziness, weakness, postexertional malaise, depression, hair loss, loss or change in taste and/or smell, gastrointestinal symptoms, cough, nausea and vomiting, sweating, palpitations, intermittent fever, chest pain/discomfort, joint pain, body aches, sleep disturbance, memory loss, hearing loss or tinnitus, anxiety, posttraumatic stress, reduced pulmonary capacity, skin rash, and allergic reactions [6-10]. More severe post–COVID-19 condition has been associated with worse well-being and quality of life, worse perceived overall health [11], difficulties in performing daily tasks [6,8,9,11], reduced ability to work [6,12], and social and family life impairment [12]. Post–COVID-19 symptoms (most commonly fatigue) and lower quality of life have been found in both hospitalized and nonhospitalized survivors of COVID-19 at follow-up 2 years after symptom onset, although in significantly lower proportions compared to the 6-month follow-up [13,14].

A number of risk factors for post–COVID-19 condition have been identified, with disease severity being a major factor. COVID-19 severity levels have been defined in clinical guidelines by the World Health Organization [15,16] and the National Institutes of Health, United States [17], as nonsevere and severe COVID-19. Nonsevere COVID-19 includes asymptomatic or presymptomatic infection [17], mild illness (symptoms of COVID-19, but without viral pneumonia or hypoxia [15,16]), and moderate illness (clinical signs of pneumonia but no signs of severe pneumonia, including oxygen saturation [SpO_2_]≥90% [15,16] or SpO_2_≥94% on room air [17]). Severe COVID-19 is defined by clinical signs of pneumonia (fever, cough, and dyspnea) plus one of the following: oxygen saturation <90% on room air; severe pneumonia; signs of severe respiratory distress [16] or by SpO_2_<94% on room air, arterial oxygen pressure [PaO_2_]/fraction of inspired oxygen [FiO_2_]<300 mm Hg, a respiratory rate >30 breaths/min, or lung infiltrates >50% [17]; it requires hospitalization and oxygen therapy. Critical COVID-19 is defined by acute respiratory distress syndrome, sepsis, septic shock, or other conditions that require mechanical ventilation (invasive or non-invasive) or vasopressor therapy [16] or respiratory failure, septic shock, and/or multiple organ dysfunction [17]; it requires intensive care unit (ICU) treatment. Severe COVID-19 disease and hospitalization have been considered as key risk factors for post–COVID-19 condition across studies [8,18-20]. Any person infected with SARS-CoV-2 can experience long-term postacute COVID-19 effects, but the proportions are significantly higher among patients admitted to hospital with severe and critical COVID-19 disease. The global estimated prevalence of post–COVID-19 condition is 34% in nonhospitalized patients and 54% in hospitalized patients [2]. A systematic review and meta-analysis estimated the prevalence of any post–COVID-19 condition symptom at 50.6% among cohorts recruited in a community setting; 66.5% among cohorts recruited in a hospital setting; and 73.8% among cohorts recruited in the ICU setting [8]. A systematic review found that studies including more hospitalized participants or more patients in ICU tended to report higher prevalence estimates: 0% to 67% in studies with 10% hospitalized participants, and 5% to 93% in studies in which all participants were hospitalized; 34.9% in studies with <5% of their samples admitted to ICU, and 48.8% in studies in which 10% or more of the sample were admitted to ICU [18]. Among hospitalized patients, those who had been more severely ill during their hospital stay had more severely impaired pulmonary diffusion capacities and abnormal chest imaging manifestations, depression or anxiety, and fatigue or muscle weakness [19]. After 2 years of symptom onset, the physical health and health-related quality of life of hospitalized survivors of COVID-19 were still poorer than those of the control population [13].

Other risk factors for post–COVID-19 condition are older age [2,10,11,18], female gender [2,10-13,19,20], preexisting chronic diseases (diabetes, hypertension, cardiovascular disease, respiratory disease, liver disease, kidney disease, and immunological disorder or allergy) [2,12,18,20], obesity [2,11,12,18], and nonvaccination status prior to infection [20].

### Long-Term Positive Psychosocial Effects of COVID-19

While medical and public health experts and the scientific community have mostly focused on the negative short- and long-term effects of COVID-19, few studies have examined the positive effects of the COVID-19 pandemic. Alongside the preponderant negative effects, positive psychological and social outcomes have been identified, including positive changes in the lives of adolescents as a whole and in the domains of mental health and well-being and improved family relationships [21-23]; positive effects in the lives of adults related to more family time, work flexibility, and calmer life [24]; and posttraumatic growth [25-32]. Posttraumatic growth is a positive psychological outcome occurring as a result of struggling with highly challenging life circumstances and traumatic experiences, which can be found in survivors of serious diseases [33]. Experiencing the pandemic and the severe COVID-19 disease can challenge the adaptive resources of individuals, and “life-changing” psychological shifts in thinking and relating to the world and the self can arise, contributing to a deeply meaningful personal process of change in the posttraumatic growth domains of appreciation of life; relating to others; personal strength; new possibilities; and spiritual, existential, or philosophical change [33]. Indeed, aspects of posttraumatic growth in relation to the experience of the COVID-19 pandemic have been identified in the general population [25] and in persons infected with SARS-CoV-2 with various levels of disease severity and place of treatment [25-30], including hospitalized survivors of severe or critical COVID-19 [27,28,31,32]. Higher levels of posttraumatic growth have been associated with a higher severity of COVID-19 acute disease [27]. COVID-19–related posttraumatic growth has been found to coexist with negative psychological trauma-related outcomes such as anxiety, depression [27,31], and posttraumatic stress disorder [25-28,31].

The coping resources available to individuals have an important role in the ability to cope with trauma and bring about positive change. Coping resources are personal, social, or other resources for managing stress and coping with adversity, which interact with coping processes [34,35]. Personal resources found to be associated with higher levels of COVID-19–induced posttraumatic growth included optimism, self-compassion, self-transcendence, religiosity/spirituality, purpose in life, agreeableness, self-efficacy, sense of coherence [25], self-esteem [25,28], and resilience [25,26]. Social support has been proven to be a significant protective factor for the development of posttraumatic growth among the general population and survivors of COVID-19 [25-30,32], with family and friends being the most important source of social support [25,30]. Financial safety has been negatively associated with posttraumatic growth [29]. In a qualitative study of females with post–COVID-19 condition, coping with the disease involved individual resources (cognitive, emotional, and spiritual), social resources (social support from family, friends, coworkers, and web-based support groups), and health systems resources (guidance and treatment by counselors and medical professionals), and insufficient financial resources have been identified as a main challenge [36].

### The Impact of the COVID-19 Pandemic on Central and Eastern Europe

Europe has been the epicenter of the pandemic several times in the period 2020-2022 and has reported the largest cumulative number of COVID-19 cases among WHO regions [1]. The countries in Central and Eastern Europe have been among the most heavily affected, which has been associated with death rates and excess mortality among the highest in the world, alongside very low vaccination rates. As of January 2024, the cumulative total reported COVID-19 deaths per 100,000 population were 556 in Bulgaria (second in the world), 460 in Croatia (seventh in the world), 389 in Slovakia (12th in the world), and 355 in Romania (15th in the world) [1]. In Europe, the countries most affected by excess mortality during the second wave (autumn to winter 2020) have been in Central and Eastern Europe (Czechia, Slovakia, Bulgaria, and Poland). In the spring of 2021, the highest mortality rates were recorded in Bulgaria (period average 168% and period maximum 202%), Romania (period average 163% and period maximum 230%), and Slovakia (period average 153% and period maximum 193%) [37]. Peaks in COVID-19 hospitalizations as high as 20,529 patients in Romania on October 26, 2021, and 10,355 patients in Bulgaria on April 11, 2021, were registered [38]. The number of patients with COVID-19 in ICUs per million reached 117,81 in Bulgaria (April 18, 2021), 112,17 in Slovakia (February 27, 2021), and 96,75 in Romania (November 7, 2021) [39]. Bulgaria, Romania, Slovakia, and Croatia have the lowest percentage of the total population vaccinated with at least 1 dose of a COVID-19 vaccine in the European Union—30% in Bulgaria, 42% in Romania, 52% in Slovakia, and 57% in Croatia [1].

In light of the repercussions of COVID-19, there is a strong need to determine pandemic and postpandemic challenges and effects at the individual, family, community, and societal levels. To advance this field, this study focuses on post–COVID-19 health and psychosocial effects, which have long-lasting impacts on the physical and mental health and quality of life of a large proportion of survivors extending beyond the end of the COVID-19 pandemic. This research complements the existing literature by examining both the negative (post–COVID-19 condition) and positive (posttraumatic growth) health and psychosocial long-term post–COVID-19 effects, their interrelationships, and their associations with the resources available to the individual to cope with stress and trauma. The study brings to the knowledge of the impact of the pandemic in the understudied region of Central and Eastern Europe, characterized by death rates and excess mortality among the highest in the world alongside very low vaccination rates associated with mistrust in vaccine benefits; mistrust of government, medical system, and medical staff; and mistrust in information from official sources [40-42]. The limited qualitative evidence regarding the outlined research topics is addressed by this study by adopting a qualitative approach that will deepen our understanding of the subjective experiences of survivors and provide valuable insights into personal meanings and the ways in which they are shaped by the specific sociocultural context and local epidemic-related situations in the studied Central and Eastern European countries. To our knowledge, this is the first study to qualitatively examine and cross-nationally compare the experiences of survivors of severe and critical COVID-19 throughout the acute and postacute period.

### Objectives

The aim of the study is to qualitatively explore the experiences of adult survivors of severe or critical COVID-19 throughout the acute and postacute period in 5 Central and Eastern European countries: Bulgaria, Slovakia, Croatia, Romania, and Poland. The study objectives are as follows: (1) to gain insight into the negative long-term post–COVID-19 effects experienced by survivors—post–COVID-19 condition (physical and mental health symptoms, cognitive symptoms, and posttraumatic stress) and its impact on quality of life (diminished functional ability, difficulties in social functioning, etc); (2) to gain insight into the experienced positive long-term post–COVID-19 effects—posttraumatic growth; (3) to understand the role of survivors’ coping resources, including personal (eg, self-efficacy, resilience, hope, optimism), social (eg, social support), and other (eg, financial) coping resources; and (4) to understand the role of the local sociocultural context and the local epidemic-related situations in the participating countries.

## Methods

### Study Design

This research is designed as an international qualitative study, adhering to the COREQ (Consolidated Criteria for Reporting Qualitative Research) guidelines.

### Theoretical Framework

The general theoretical framework of the study is the biopsychosocial model of health and illness [43], which conceptualizes our understanding of the COVID-19 disease as a result of the interaction of biological, psychological, and social factors.

The study follows the methodological orientation of qualitative thematic analysis [44-46] and the approach of reflexive thematic analysis [46], emphasizing the subjectivity of research and the researcher’s active role in coding and theme generation. Themes are produced by the researcher(s) through systematic analytical engagement with the dataset and through personal positioning and metatheoretical perspectives. We adopt an experiential reflexive thematic analysis perspective focusing on participants’ experiences and sense-making. The study has an inductive orientation, which is understood as “grounded” in data, considering that pure induction is impossible and data analysis is always underpinned by theoretical assumptions [46].

Our analytical approach includes a 2-stage data analysis. In stage 1, thematic analysis of each national dataset will be conducted and a national thematic map for each country’s data will be produced, allowing for a meaningful discussion of findings and their implications for policy and practice at the national level. In stage 2, the national analyses will be collated and an international thematic map will be produced, allowing for highlighting cross-national differences, underlying epidemic-related and sociocultural factors, and drawing conclusions about the region of Central and Eastern Europe.

### Study Site

This international study is conducted in 5 countries in Central and Eastern Europe: Bulgaria, Slovakia, Croatia, Romania, and Poland. The sampling frame used across countries comprises lists of discharged patients with severe or critical COVID-19 who have given their consent to be contacted, including lists from major public and private hospitals, rehabilitation centers, and recreational facilities that have treated patients with COVID-19 in the respective country, as well as from individual health care practitioners and lists created by the national research teams based on their private contacts, faculty, and students.

### Study Duration

The study duration is 3 years (January 2022 to December 2024). Initially, the study started with 4 participating countries (Bulgaria, Slovakia, Croatia, Romania), and Poland was associated with the study at a later stage (in November 2022).

### Research Team and Reflexivity

The implementing partners who are responsible for conducting the study in the 5 countries are the Department of Psychology, Institute for Population and Human Studies at the Bulgarian Academy of Sciences (Sofia, Bulgaria); the Centre of Social and Psychological Sciences, Slovak Academy of Sciences (Bratislava, Slovakia); the Department of Psychology, Faculty of Humanities and Social Sciences, University of Zagreb (Zagreb, Croatia); the Department of Psychology, Babes-Bolyai University (Cluj-Napoca, Romania); and the Faculty of Health Science, Ludwik Rydygier Collegium Medicum in Bydgoszcz, Nicolaus Copernicus University in Toruń (Bydgoszcz, Poland). The members of the national research teams are psychologists holding academic positions in the above-listed institutions and PhD students in psychology. All researchers involved in data collection received training in qualitative data collection according to a standardized training protocol developed specifically for the study.

### Participant Selection

Participants are selected through purposive sampling and snowball sampling. The purposive sampling strategy aims to achieve maximum variation and capture a wide range of perspectives and experiences. Participants’ characteristics of interest include gender, age, country of residence, city of residence, need for ICU treatment, length of postdischarge period, and chronic diseases.

In this study, severe and critical COVID-19 disease is defined in accordance with WHO and National Institutes of Health clinical guidelines and their clinical classifications of COVID-19 severity [15-17], using the clinical judgment of the patient’s condition as requiring hospitalization (severe COVID-19 disease) or ICU care (critical COVID-19 disease) according to WHO guidance as the main criterion. Participants have been enrolled based on self-reported severe or critical COVID-19 disease.

The eligibility criteria for participation in the study include the following inclusion and exclusion criteria:

- Inclusion criteria: aged 18 years or older; tested positive for SARS-CoV-2; has had evidence of pneumonia; has been hospitalized (for severe COVID-19) or has been hospitalized and admitted to the ICU (for critical COVID-19); a resident of Bulgaria, Slovakia, Croatia, Romania, or Poland; and provided consent to participate in the study
- Exclusion criteria: treated for severe COVID-19 in a country other than the country of residence, member of the family of a participant, or unwillingness to comply with the study protocol

The national research teams approach participants in several different ways. Eligible participants who have given their consent are approached through the health care system, including hospitals, rehabilitation centers, recreational facilities for recovering from severe COVID-19, and individual health care practitioners. A group of participants is recruited through private contacts of the research team members and faculty and students in the participating research institutions. Another group is approached through advertisements on social media. The snowball technique is also used, and participants have been asked to invite other survivors of severe and critical COVID-19 to participate in the study. The participants are contacted via telephone or email.

### Sample Size

The sample size of this study has been determined based on the suggestion of Braun and Clarke [47] that qualitative studies of experiences and influencing factors using interviews and a thematic analysis approach require a sample size of 15-30 participants in order to convincingly demonstrate patterns across the dataset while retaining a focus on individual experiences. The sample size is guided also by the concept of information power in qualitative interview studies [48], which considers that a broader study aim, a study supported by limited theoretical perspectives, and a cross-case analysis require a larger sample size. Based on the adequacy of the sample size for stage 1 thematic analysis, the target sample size has been decided to include 30 participants in each participating country.

### Setting

Across countries, participants are sampled from public and private hospitals, rehabilitation centers, recreational facilities, and individual health care practitioners’ practices, as well as from research and higher education institutions accessible to the researchers. Additionally, some participants are sampled from the general population after responding to invitations to participate in the study posted on social media or promoted by the researchers and their contacts. Data are collected through in-depth, semistructured interviews, conducted one on one by the members of the national research teams in person, through videoconferencing or by telephone. The in-person interviews are conducted in counseling rooms or at the premises of the research institutions.

### Data Collection

The research methodology and study documentation have been developed in English and then translated into the national languages of the participating countries: Bulgarian, Slovak, Croatian, Romanian, and Polish. The method for qualitative data collection is in-depth, semistructured interviews, conducted after hospital discharge. The first 4 conducted interviews in each country serve as a pilot study for testing the study instrument and procedures. Based on the pilot study, revisions to the methodology have been made.

Instruments include a specifically designed for the study semistructured questionnaire (interview guide; Multimedia Appendix 1) and a sociodemographic questionnaire, which have been prepared based on a literature review. The topics in the semistructured questionnaire follow the experiences of survivors throughout the trajectory of the disease, from preinfection COVID-19 attitudes to first symptoms, hospitalization, recovery, and postdischarge adjustment. The interview guide covers various aspects such as vaccination attitudes and decision-making; illness experience; aggravating of existing chronic conditions; difficulties with access to testing, treatment, and hospitalization; hospitalization experience; communication with medical personnel; coping resources and coping strategies; social support; postdischarge experiences; impaired quality of life; and posttraumatic growth. The sociodemographic questionnaire collects data on gender, age, country of residence, city of residence, ethnicity, marital status, having children, educational level, profession/occupation, length of hospitalization, ICU treatment, length of postdischarge period, and chronic diseases.

The duration of the interviews depends on the natural flow of the conversation, with each participant being given sufficient time to fully share their experiences. To ensure accurate and complete data representation, an audio recording of the conversation is made after receiving permission from the participant. In the case of videoconferencing, only the audio recording of the meeting is used.

### Ethical Considerations

Ethics approval for the study has been obtained from the Ethical Committee of the Institute for Population and Human Studies—Bulgarian Academy of Sciences (ethical approval number PD-2-140/15.08.2022).

Participants give their informed consent about their voluntary participation in the study. They are provided with the opportunity to withdraw their consent and terminate their participation at any time. An information sheet is part of the ethical documentation along with the informed consent form. It provides information about the study and explains the procedure, voluntary participation, the right to withdraw from the study, anonymity and confidentiality of participant information, potential benefits and risks associated with study participation, and the use of collected data only for scientific purposes.

Collected qualitative data are anonymized, with all potentially identifying information removed. Demographic information and participant-identifying data are organized in separate files. Demographic information and qualitative data are analyzed and reported in aggregate form only.

Participants receive no compensation for their participation in the study.

### Data Analysis

The audiotaped interviews are transcribed verbatim using the automated transcription package of the NVivo software (Lumivero) for qualitative data analysis, which supports transcription in the national languages of the partners. The transcriptions are then checked by the researchers for accuracy and are corrected where needed.

Data are analyzed through reflexive thematic analysis [44-46]. The analytic process involves searching for meanings and patterns in the data, generating codes, grouping codes into themes, and producing a thematic map—a complete conceptualization of the patterns in the data and the connections between them. The 6-phase process includes familiarizing with the data; generating initial codes; generating initial themes; reviewing potential themes; defining and naming themes; and producing the report. A total of 2-4 researchers code and compare the data in the different countries. The analysis is assisted by NVivo software for qualitative data analysis.

Data analysis is conducted in 2 stages. Stage 1 includes thematic analysis at the national level conducted by the national research teams on their national dataset in their national language. Stage 2 includes the translation of the national thematic maps into English and the integration of the national-level results of the 5 participating countries. The process will involve discussions of data and results between the national teams.

## Results

### Recruitment and Data Collection

Recruitment of participants and qualitative data collection (pilot study) started at the end of August 2022 for the main participating countries. Poland joined the study as an associate partner at a later stage (at the end of November 2022) and started recruiting participants and collecting data in January 2023.

As of the publication of this paper, data collection is complete in all countries. The total international sample includes 151 survivors of severe and critical COVID-19 in the 5 Central and Eastern European countries: Bulgaria (n=33, 21.8%), Slovakia (n=30, 19.9%), Croatia (n=30, 19.9%), Romania (n=30, 19.9%), and Poland (n=28, 18.5%). In Poland, the sample includes fewer than 30 participants, as 4 participants have withdrawn their consent to participate in the study.

### Data Analysis and Expected Results

Transcription is complete in all countries. National-level qualitative thematic analysis is currently underway. The Slovak [49] and Romanian [50,51] teams have published research articles based on the analysis of their national datasets, presenting findings on posttraumatic growth and vaccine decision-making. Cross-national analysis has started in 2024. The results will be submitted for publication in the third and fourth quarters of 2024.

## Discussion

### Principal Findings

The study aims to gain a deeper understanding of the experiences of survivors of severe and critical COVID-19 throughout the acute and postacute period in 5 Central and Eastern European countries: Bulgaria, Slovakia, Croatia, Romania, and Poland. The results of the research will be presented in national thematic maps of the experiences of people who survived severe COVID-19 and in an international thematic map delineating the experiences of survivors in the region of Central and Eastern Europe. Thematic maps will visually represent the interconnected themes identified in the analysis and improve our understanding of the complex relationships between illness experience, negative and positive long-term health and psychosocial post–COVID-19 effects such as the post–COVID-19 condition, impaired quality of life, and posttraumatic growth, influencing factors such as coping resources and processes, and the specific sociocultural context and local epidemic-related situations that shape the experiences and impact the health outcomes of survivors of COVID-19.

Published results based on the analysis of Slovak [49] and Romanian [51] data provide insight into experiences of posttraumatic growth that have occurred through a reassessment of priorities and changed life perspective and appreciation of life itself and loved ones, and are built on effective coping strategies, self-care, inner strengths (such as optimism, determination, fighting difficulties attitude), and gratitude, highlighting pathways through which health adversities have induced enduring positive changes across cognitive, emotional, behavioral, and relational domains. Findings on survivors’ perspectives on vaccination from Romania [50] increase our understanding of vaccine decision-making, underscoring severe illness as a factor for vaccine acceptance and vaccine adverse reactions as a factor for hesitancy, and the role of influences from trustworthy relationships and general disbelief and conspiracy theories in the decision-making process.

Ongoing research will shed further light and advance scientific knowledge on the acute and prolonged forms and physical and mental health symptoms of the coronavirus disease, the emergence and coexistence of positive psychological outcomes in the aftermath of psychological trauma and functional impairment, the multifaceted subjective experiences of patients, the diverse mobilized coping resources during the fight with severe COVID-19, survivors’ needs for psychosocial support through the recovery process, and the impacts of the specific sociocultural contexts in the Central and Eastern European region. Based on the results, we will make recommendations that can inform the development of socioculturally appropriate tailored approaches to providing health and psychosocial support for coping with the post-COVID-19 condition and associated difficulties in different life domains, and the implementation of interventions to facilitate posttraumatic growth.

### Limitations

The study limitations could include the possibility of sampling bias, as individuals having multiple pronounced long-term post–COVID-19 symptoms may be more likely to participate due to relating to the study and wanting to share their experiences. On the other hand, individuals with higher levels of post–COVID-19 trauma and posttraumatic stress disorder may be less likely to participate due to experiencing retraumatization while recalling painful experiences. This could potentially lead to overrepresentation and underrepresentation of specific groups and bias within the study’s findings. Self-selection bias is possible to arise due to using recruitment strategies such as advertisements about the study on social media. To minimize selection biases, a purposive sampling approach aiming at maximum variability and specific inclusion and exclusion criteria are implemented. Participants are recruited through diverse recruitment methods, and data are collected on their motivations for participation and nonparticipation. Collecting data on preillness and illness experiences in the postacute period postulates the retrospective nature of the study and the possibility for recall bias. A strategy to mitigate this bias is to achieve diverse lengths of the postdischarge period, ranging from participants who have been recently released from hospital to participants who have been infected and treated during the first and second waves of the COVID-19 pandemic.

### Conclusions

This study protocol outlines an international qualitative investigation into the experiences of survivors of severe and critical COVID-19 of acute illness and long-term negative and positive post–COVID-19 health and psychosocial effects in the understudied region of Central and Eastern Europe. It emphasizes the importance of a deeper understanding of the ongoing health and psychosocial challenges survivors face and what helps them cope with these challenges and, in some cases, thrive. The results will add knowledge that can inform health care practice in hospitals and rehabilitation services, informal caregiving, and provision of psychological support to survivors of COVID-19 and ultimately improve the health and psychosocial outcomes of adult survivors of COVID-19. In the aftermath of a global pandemic, this research will be crucial for evaluating its overall impact and multifaceted implications and for informing future pandemic preparedness.

## Appendix

Multimedia Appendix 1 Interview guide.

## Abbreviations

COREQ: Consolidated Criteria for Reporting Qualitative Research
FiO2: fraction of inspired oxygen
ICU: intensive care unit
PaO2: arterial oxygen pressure
PASC: postacute sequelae of SARS-CoV-2 infection
SpO2: oxygen saturation
WHO: World Health Organization

## Data Availability

The datasets generated and analyzed during this study are not publicly available due to the nature of the research containing potentially identifying and sensitive patient information but are available from the corresponding author on reasonable request.
