# Supplementary material for: Long-Term Post–COVID-19 Health and Psychosocial Effects and Coping Resources Among Survivors of Severe and Critical COVID-19 in Central and Eastern Europe: Protocol for an International Qualitative Study
Source: JMIR Res Protoc. 2024 Sep 30;13:e57596. doi: 10.2196/57596 (PMC11474134; doi:10.2196/57596)
Supplement: Multimedia Appendix 1 [file resprot_v13i1e57596_app1.docx]

**INTERVIEW GUIDE**

1. Please introduce yourself briefly.
2. What were your thoughts and feelings at the outbreak of COVID-19 in your country, and how have they changed over the course of the pandemic?
3. What did you think and feel about COVID-19 before you got sick?
4. What did your family, friends and people in your social network think and feel about COVID-19 before you got sick?
5. Have you and people in your social network been vaccinated against COVID-19?
6. Have you had family or friends who have been seriously ill from COVID-19 (or died from COVID-19)?
7. What do you think about the COVID-19-related actions of the government, health authorities, and medical professionals in your country?
8. In your opinion, what are the prevailing COVID-19-related attitudes of people in your country?
9. How did you become infected with COVID-19 and how did your illness progress?
10. What were your COVID-19 symptoms?
11. How were you diagnosed with COVID-19? How were you hospitalized? Were there problems such as not enough tests/difficult access to testing, overwhelmed hospitals/difficult access to hospital? Were you immediately hospitalized at a nearby hospital?
12. What was your health status when you got infected? Did your chronic conditions play a role in the course of your illness?
13. What were your thoughts and feelings during the contact with the infected person, first symptoms, diagnosis, and hospitalization?
14. Did other members of your family (friends) also got infected with COVID-19 at the same time and how did their illness progress? (e.g. mild symptoms, hospitalization, etc.)
15. If other members of your family were also infected at the same time, how did this affect your family life?
16. Did you feel supported by your family and friends? How did they support you?
17. What were your experiences during your stay in the hospital?
18. What was your treatment? Did your chronic diseases aggravate your condition and require special treatment? Were you treated in an intensive care unit?
19. How did your illness progress during your stay in the hospital? How long were you hospitalized?
20. What were your thoughts and feelings during your stay in the hospital?
21. Did you have any unusual experiences during your oxygen therapy (ventilation) like dreams, dream-like sensations, fantasies, nightmares, hallucinations, or others?
22. What were your relationships with other patients and with the medical personnel like? (Were there other members of your family hospitalized in the same hospital?)
23. Did you feel supported by your family and friends? How did they support you?
24. Overall, how satisfied are you with your stay in the hospital? What are you most satisfied with? What could have been better?
25. What have been your experiences in the period since you were discharged from the hospital?
26. How was your discharge from the hospital?
27. How did your family react to the way you looked and felt at your hospital discharge? (e.g. reactions to unhealthy looks, massive weight loss, weakness, etc., child/adolescent reactions)
28. How would you describe your current health status? (What about your chronic conditions?)
29. Have you had symptoms that persist from the acute illness? Please list them. How long have they persisted? (e.g. physical and mental health symptoms, cognitive symptoms, post-traumatic stress, etc.)
30. Have you had any new symptoms? Please list them. How long have they lasted?
31. Have you had follow-up visits with a doctor after you were discharged from the hospital? Have you sought medical/psychological help for your post-Covid symptoms? Are you currently receiving treatment for them?
32. Are you able to do the activities you did before your COVID-19 disease? (e.g., physical activity/walking/climbing stairs, housework, childcare, etc.)
33. Do you feel able to go back to work (school/university)? (When were you able to go back to work/school/university?) Are you able to do your work-related activities as you did before your COVID-19 disease?
34. How is your family life in the post-discharge period? (e.g. relationships, housework, financial difficulties, etc.)
35. How is your social life in the post-discharge period? (e.g. relationships/meeting with friends, attending public places/events, hobbies, sports, etc.)
36. Do you feel supported by your family and friends? How do they support you?
37. Have you sought help/psychological support/communication with other COVID-19 survivors/support groups?
38. What are your thoughts and feelings in the period after hospital discharge? Are you optimistic or pessimistic?
39. Has anything changed for you and your life after you survived severe/critical COVID-19? Do you see yourself and your life differently now? What have you learned from this experience? (e.g. aspects of post-traumatic growth).
40. What helps you cope with the long-term consequences of the disease? (Coping resources: e.g. your personal qualities/the kind of person you are, support from family and friends, availability of post-Covid treatment, financial stability, etc.).
41. **(For patients not vaccinated prior to COVID-19).** Would you get vaccinated if you knew what severe COVID-19 was like? Do you think you would have avoided severe COVID-19 and hospitalization if you had been vaccinated? Are you vaccinated now? Do you intend to get vaccinated?
42. Is there anything else you would like to add about COVID-19 and your experiences that was not covered in the questions?

**Thank you for participating in this interview!**
